# Solvent-annealing-induced microphase separation in polyether polyurethane: a small-angle X-ray scattering study

**DOI:** 10.1107/S1600576725001633

**Published:** 2025-03-19

**Authors:** Shanshan Wang, Jiayao Song, Keping Chen, Mark Julian Henderson, Qiang Tian, László Almásy

**Affiliations:** a State Key Laboratory of Environment-Friendly Energy Materials, School of Materials and Chemistry Southwest University of Science and Technology Mianyang621010 People’s Republic of China; b Institute of Chemical Materials China Academy of Engineering Physics Mianyang621900 People’s Republic of China; c Institute for Energy Security and Environmental Safety, HUN-REN Centre for Energy Research, Konkoly Thege Miklos út 29-33, Budapest1121, Hungary; Australian Centre for Neutron Scattering, ANSTO, Australia

**Keywords:** polyurethane, solvent annealing, microphase separation, small-angle X-ray scattering, SAXS

## Abstract

This study investigated the effects of solvent vapor annealing on the microphase separation structure of polyurethane (PU) using small-angle X-ray scattering (SAXS) and complementary techniques. Solvent annealing, as an alternative to thermal annealing, offers a lower-temperature method to refine the microstructure of PU. We examined the impact of methyl ethyl ketone (MEK), acetone and toluene vapors on a commercial polyether PU, focusing on changes in microphase structure, adsorption kinetics and thermal stability. The SAXS data, analyzed by a polydisperse hard-sphere model, indicated that the degree of phase separation increased upon solvent annealing, and the order of influence exerted by the solvent vapors on the microphase structure follows MEK > acetone > toluene. The *in situ* variable-temperature SAXS results showed that the solvent-annealed sample had superior thermal stability to the quenched sample. Compared with high-temperature annealing, solvent annealing induced a higher degree of phase separation but did not lead to significant growth of the hard-urethane-segment-rich domains. These findings provide valuable insights into optimizing solvent annealing processes, allowing for advanced applications of PUs where excessive heat may lead to degradation or other undesirable changes.

## Abstract 2

Small-angle X-ray scattering was applied to polyether polyurethane to investigate the effects of solvent vapor annealing on the microphase separation. Among the solvents studied, methyl ethyl ketone induced the greatest degree of phase separation in polyurethane compared with the thermally annealed state.

## 1. Introduction

The microphase-separated structure of polyurethane (PU), as described by the dispersion of hard-urethane-segment-rich (HS) domains, which act as physical cross-linking points in soft-polyester/polyether-segment-rich (SS) matrices, exerts a profound influence on its macroscopic performance (Masuda *et al.*, 1992 ▸; Qi & Boyce, 2005 ▸; Jiang *et al.*, 2012 ▸; Sakurai *et al.*, 1994 ▸). Traditionally, the microstructure of PU is modulated by thermal annealing (Nachman & Kwiatkowski, 2013 ▸; Liu *et al.*, 2020 ▸; Jayasuriya *et al.*, 1997 ▸; Kazmierczak *et al.*, 2003 ▸) and chemical modifications (Lin *et al.*, 2017 ▸; Bugrov *et al.*, 2022 ▸; Chen *et al.*, 2017 ▸; Prisacariu, 2011 ▸; Chu *et al.*, 1992 ▸, Leung & Koberstein, 1985 ▸). It was found that the microphase separation in poly(tetra­methyl­ene oxide)-based PU is more complete compared with poly(ethyl­ene oxide)-based PU, and the microphase separation kinetics proceed significantly faster with increasing soft-segment length (Chu *et al.*, 1992 ▸). Yanagihara *et al.* (2015 ▸) reported that the increase of annealing temperature to 145°C leads to a rise in size and volume fraction of HS domains. Additionally, the elastic moduli of the thermally annealed samples were all greater than those of the quenched ones. Jiang *et al.* (2012 ▸) found that thermal annealing at 180°C for 1 h increases the 100% elongation modulus from 9.9 to 10.9 MPa in their study of PU/multi-walled carbon nanotube nanocomposites. However, in the case of PU-containing composites or for devices that are sensitive to temperature, such as polymer-bonded explosives and electronic devices that utilize PU (Armstrong & Mang, 2021 ▸; Li *et al.*, 2019 ▸), the application of high-temperature annealing is not suitable.

Solvent annealing is a process that involves the treatment of polymer films in a solvent vapor atmosphere, allowing the polymer to obtain a refined microstructure and enhanced macroscopic properties. Unlike thermal annealing, solvent annealing does not require high temperatures. The principle behind solvent annealing is the use of a solvent vapor that has good compatibility with the polymer matrix. This increases macromolecular mobility due to solvent penetration, thereby achieving a new thermodynamic equilibrium at room temperature (Ge *et al.*, 2021 ▸; Xu *et al.*, 2024 ▸). This approach has been successfully applied to a variety of polymetric materials, enabling the tailoring of their phase separation, crystallinity and other characteristics. Dou *et al.* (2018 ▸) found that solvent annealing using *N*-methyl­pyrrolidone significantly changes the morphological characteristics of chalcogenide films by eliminating surface defects and promoting grain growth. Hu *et al.* (2022 ▸) reported that using CS_2_ to anneal organic solar cell devices enhances molecular aggregation and crystallinity, leading to a more distinct phase separation in the blends. Ding *et al.* (2022 ▸) found that, after toluene solvent treatment, the tensile strength of PU samples increases from 27 to 51 MPa, and the elongation at a break increases from 330 to 525%. Shao *et al.* (2016 ▸) found that exposing polybutene-1 to chloro­form vapor can accelerate the transition from crystal form II to form I, but this process also results in a reduction in the overall crystallinity of the material. Tseng *et al.* (2017 ▸) studied the effects of solvents annealing on the morphological evolution of polystyrene (PS) microspheres spread on the surface of polymethyl methacrylate (PMMA) film. Their findings revealed that the choice of solvent has a significant impact on the resulting microstructure. Specifically, the use of cyclo­hexane as the annealing solvent causes the PS microspheres to form disc-like structures on the PMMA film. In contrast, the use of toluene leads to the formation of anisotropic PS particles embedded within the PMMA film. All of this work has shown great potential in tuning the microstructures and properties of polymetric materials, such as segmented polymers and polymer composites. However, the underlying mechanisms governing the solvent–polymer interactions during the annealing process are not yet fully understood. The complex interplay between the solvent molecules and the polymer network structure, including factors such as sorption, chain mobility and interaction parameters, requires deeper investigation. A better understanding of the fundamental solvent–polymer interactions would provide valuable insights for the rational design and precise control of the final polymer microstructure and properties.

Small-angle scattering (SAS) is a powerful technique for studying the microphase structure of PUs, allowing for the determination of the size, volume fraction and spatial relationships of the HS domains (Leung & Koberstein, 1985 ▸; Bonart & Müller, 1974 ▸; Laity *et al.*, 2004 ▸; Tian *et al.*, 2018 ▸; Tian *et al.*, 2015 ▸). Our previous small-angle neutron scattering studies revealed that, on thermal–humid aging, polyester PU exhibits an increase in the interdomain distance and domain size (Tian *et al.*, 2014 ▸; Tian *et al.*, 2016 ▸). Recently, Wang *et al.* (2023 ▸) and Song *et al.* (2024 ▸) studied the effects of water sorption on polyether PU and polyurea, respectively. Their small-angle X-ray scattering (SAXS) data indicated that the water molecules can penetrate the polymer networks and disrupt the loose HS domains. The findings from these SAS studies highlight the importance of understanding the water–polymer interactions at the nanoscale. Polymers such as PU have a greater capacity to adsorb organic solvent molecules than water. Consequently, the incorporation of organic molecules by solvent annealing can lead to more significant structural changes in the polymer network. The reorganization of the polymer microstructure induced by organic solvent penetration and interaction can be monitored using SAS as a function of solvent type, concentration, annealing time and so on.

In this work, a commercial polyether PU was employed for a solvent annealing study, investigating the effects of different solvents and annealing durations. The SAXS analysis, along with complementary techniques, provided detailed insights into the structural evolution and mechanism of the PU on exposure to different solvent vapors. Our findings indicate that the strong affinity between the methyl ethyl ketone (MEK) solvent and the PU chains induced the most pronounced microphase separation in the PU samples.

## 2. Experimental

### 2.1. Material

Thermoplastic PU 1180A, in pellet form, was commercially obtained from BASF (Badische Anilin-und-Soda-Fabrik, Germany). It is a segmented copolymer containing approximately 28 wt% hard segment, prepared by the reaction of di­phenyl­methane diiso­cyanate (MDI), poly(tetra­hydro­furan)­glycol and 1,4-butane­diol (BDO) as a chain extender. The chemical structure of the repeating units of PU 1180A is shown in Fig. S1 of the supporting information. The number-average molecular weight of PU 1180A is 70 kDa (Kong *et al.*, 2020 ▸). The pellets were hot-pressed at 185°C and then immediately quenched in cold water to form a film of 0.3 mm in thickness.

### 2.2. Solvent annealing

Acetone, MEK and toluene were placed in separate containers. The quenched PU samples were cut into 20 × 10 × 0.3 mm pieces and positioned above the liquid solvent surface in the containers. After being kept in the sealed containers for 0.5, 1, 5, 10, 20 and 30 d, the solvent-annealed samples were removed and dried under vacuum to remove any residual organic solvents. In addition to the three solvents mentioned, *N*,*N*-di­methyl­formamide and tetra­hydro­furan were also selected for the solvent annealing experiments. However, we observed that the PU samples exposed to these vapors resulted in sticky gels.

### 2.3. Adsorption experiment

The cold-water-quenched (35 × 10 × 0.3 mm) samples were placed above either acetone, MEK, toluene or water. The samples were removed from the sealed beakers at specific time intervals and weighed to an accuracy of ±0.1 mg. To ensure reliability, three samples were measured for each condition and the average values were recorded.

### 2.4. Characterization

#### 2.4.1. SAXS

The SAXS measurements were carried out on a SAXSpace diffractometer (Anton Paar, Austria), operated at 40 kV and 50 mA. The intensity and position of the scattered rays were recorded by a one-dimensional Mythen2 R 1K detector (Dectris, Switzerland) with a resolution of 50 µm. The scattering intensity *I* was calculated as a function of the magnitude of the scattering vector *q*, where *q* = 4π sin θ / λ, θ is half of the scattering angle and λ = 0.154 nm is the incident X-ray wavelength. The detector was 317 mm from the sample, resulting in the *q* range 0.1−7.5 nm^−1^. For the standard SAXS measurements, a copper plate with a slit length of 20 mm was used as the sample holder. For the *in situ* variable-temperature SAXS measurements, the sample was wrapped in aluminium foil and mounted on a hot stage. The heating rate was set to 10°C min^−1^ and the sample was held at the target temperature for 5 min before data collection. The sample was heated from 20 to 140°C in 20°C intervals. An exposure time of 10 min was sufficient to give an acceptable signal-to-noise ratio. All data were normalized to the incident primary beam intensity, corrected for background scattering and desmeared using the *SAXSquant* software (version 4.1.0.7505; Anton Paar). The reduced *I*–*q* curves were fitted by the least-squares method using the *SASfit* software (version 0.94.11; Kohlbrecher & Breßler, 2022 ▸).

#### 2.4.2. FTIR and DSC

Fourier transform infrared spectroscopy (FTIR) measurements were performed using a Nicolet-6700 (Thermo Electron Co. USA) attenuated total reflection system. Data were recorded by averaging 32 scans across the wavenumber range from 4000 to 400 cm^−1^ with a resolution of 4 cm^−1^. FTIR analysis was used to probe the ratio of free C=O and hydrogen-bonded C=O species within the PU samples. The broad absorption peak from 1760 to 1660 cm^−1^ is attributed to the stretching vibration of the C=O group; the contents of ordered hydrogen bonding C=O, disordered hydrogen bonding C=O and free C=O were calculated from the peak areas according to equations (S1)–(S3) of the supporting information (Zhang *et al.*, 2020 ▸; Arunkumar *et al.*, 2023 ▸). Approximately 4.5 mg of sample was taken for calorimetric measurements using a differential scanning calorimetry instrument (DSC Q2000, TA Instruments, USA). The measurements were recorded over the temperature range 0–250°C with a scanning rate of 10°C min^−1^. The testing process was carried out under a nitro­gen atmosphere with a flow rate of 50 ml min^−1^.

## 3. Results and discussion

### 3.1. Solvent vapor adsorption

**Figure 1:**
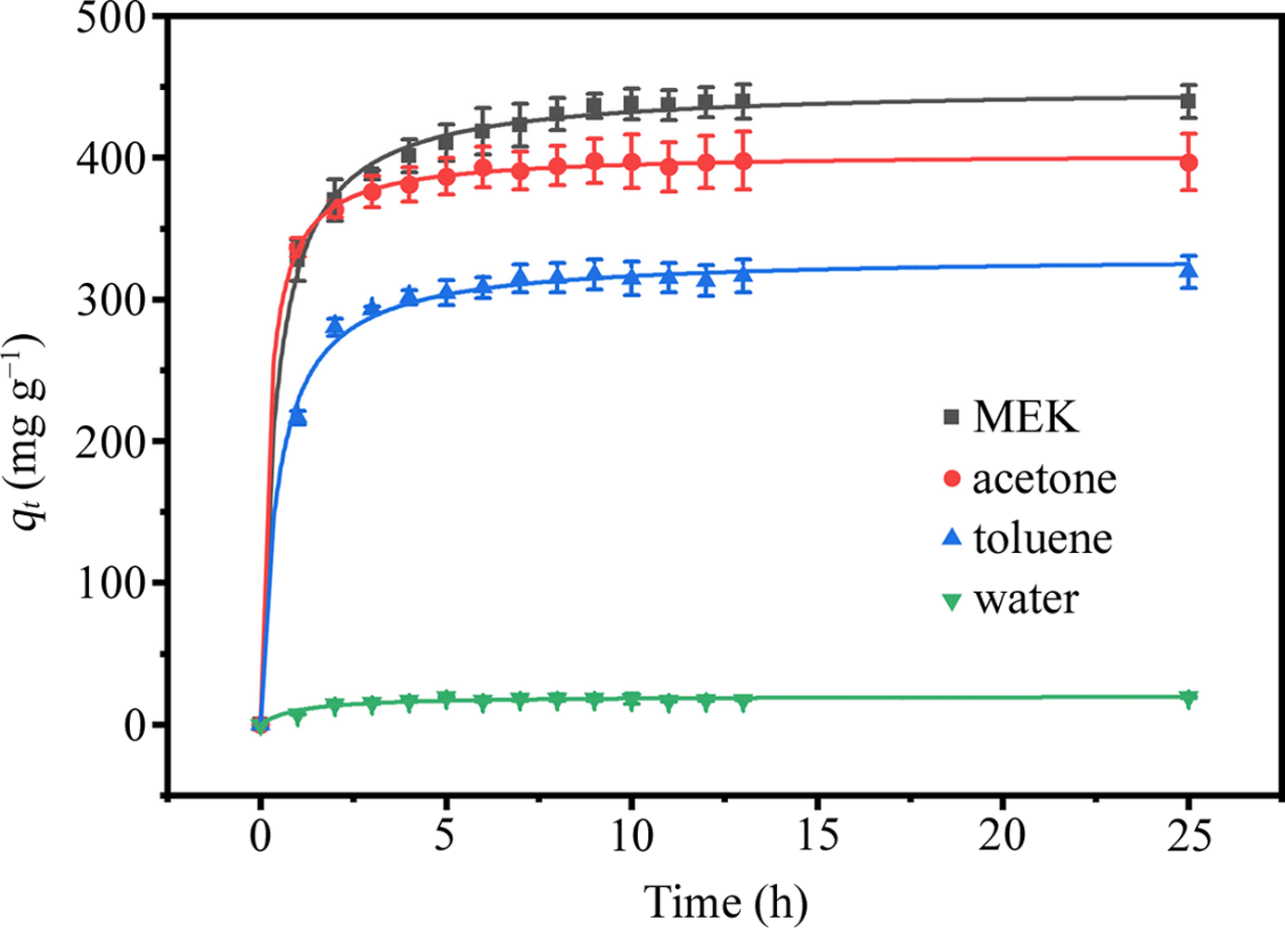
Adsorption kinetics curves of PU 1180A for MEK, acetone, toluene and water vapors.

The adsorption kinetic curves of the PU samples are shown in Fig. 1 ▸. On the basis of the height of the saturation adsorption plateau, PU 1180A exhibited the highest adsorption capacity for MEK vapor. In contrast, the adsorption capacity for water is much smaller than those for the organic vapors. The pseudo-second-order kinetics model was employed to fit the experimental data,
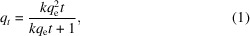
where *t* is the adsorption time, *q*_e_ is the adsorption capacity of PU to solvent at adsorption equilibrium, *q_t_* is the adsorption capacity at time *t* and *k* is the pseudo-second-order kinetics rate constant. The pseudo-second-order kinetics model fitting is in good agreement with the experimental data (Fig. 1 ▸), indicating that chemisorption plays an important role in the adsorption process. The fitted parameters are shown in Table 1 ▸. After 10 h, the calculated *q_t_* values of MEK, acetone, toluene and water are 96.0, 98.0, 95.7 and 95.8% of *q*_e_, respectively. The derived *q*_e_ of PU 1180A for these solvents followed the order MEK (450 mg g^−1^) > acetone (403 mg g^−1^) > toluene (331 mg g^−1^) ≫ water (19 mg g^−1^). Note that the rate constant for the adsorption of water vapor is the highest among the studied vapors, which is probably attributable to its predominant surface adsorption. Accordingly, the adsorption of organic solvents by PU occurred both on the surface and in the bulk.

### 3.2. Microphase structure

**Figure 2:**
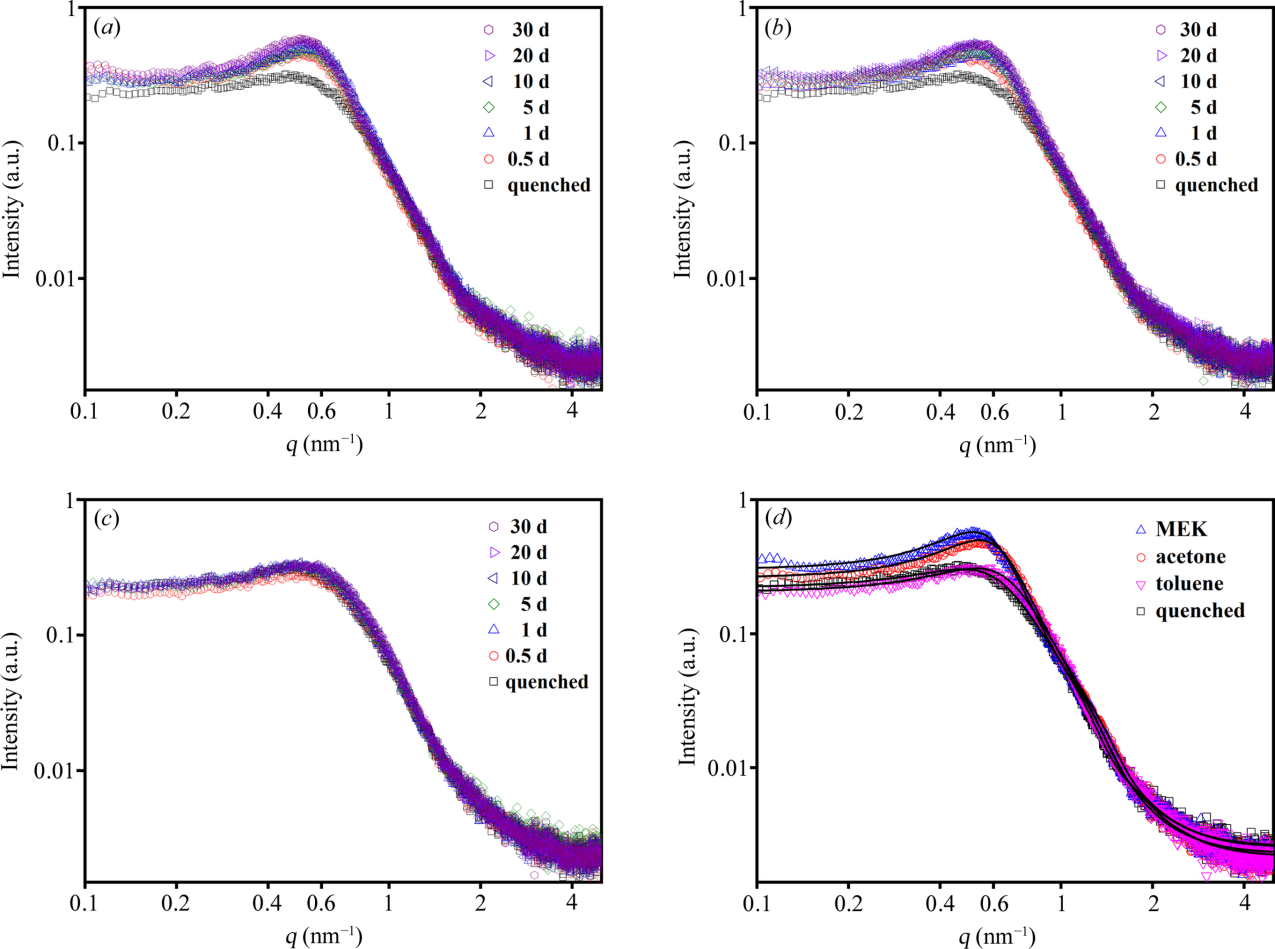
SAXS data obtained from the annealed PU 1180A with organic solvents: (*a*) MEK, (*b*) acetone, (*c*) toluene, (*d*) annealed for 30 d. The solid lines in (*d*) represent the best fits to the polydisperse hard-sphere model.

The SAXS data obtained from the solvent-annealed PU samples are shown in Fig. 2 ▸. All *I*–*q* curves show a broad peak around 0.6 nm^−1^ due to the interference scattering from the HS domains. The position of the scattering peak remained nearly constant with increasing solvent annealing time. However, the intensity of the scattering peak increased strongly at the early stage, and thereafter showed a gradually stabilizing pattern after 10 d. Samples annealed for 30 days in solvent represented a near-equilibrium structural state. Comparative analysis of the scattering patterns revealed that the MEK vapor exerted the most pronounced influence on the PU sample [Fig. 2 ▸(*d*)].

In order to extract structural information, a polydisperse hard-sphere model proposed in our previous research was employed to fit the SAXS data (Tian *et al.*, 2018 ▸; Kong *et al.*, 2019 ▸; Chen *et al.*, 2017 ▸). In this model, the scattering intensity is expressed as the product of the size-averaged form factor and hard-sphere interaction structure factor, written as
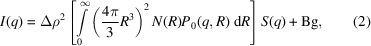

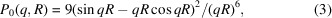

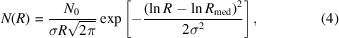

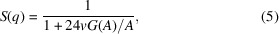
where Δρ is the scattering length density difference between the HS domains and the SS matrix; *P*_0_(*q*, *R*) is the normalized form factor of spherical HS domains; *N*(*R*) d*R* is the number density of the HS domains with sizes between *R* and *R* + d*R*; *S*(*q*) is the Percus–Yevick structure factor, applicable for hard-sphere interactions; Bg is a constant representing the scattering background; *R*_med_ is the median radius; σ is the logarithmic standard deviation, reflecting the polydispersity of the HD domains; and *A* and *G* are algebraic functions of the hard-sphere interaction radius (*R*_HS_) and hard-sphere volume fraction (*v*) (Percus & Yevick, 1958 ▸; Tian *et al.*, 2014 ▸).

**Figure 3:**
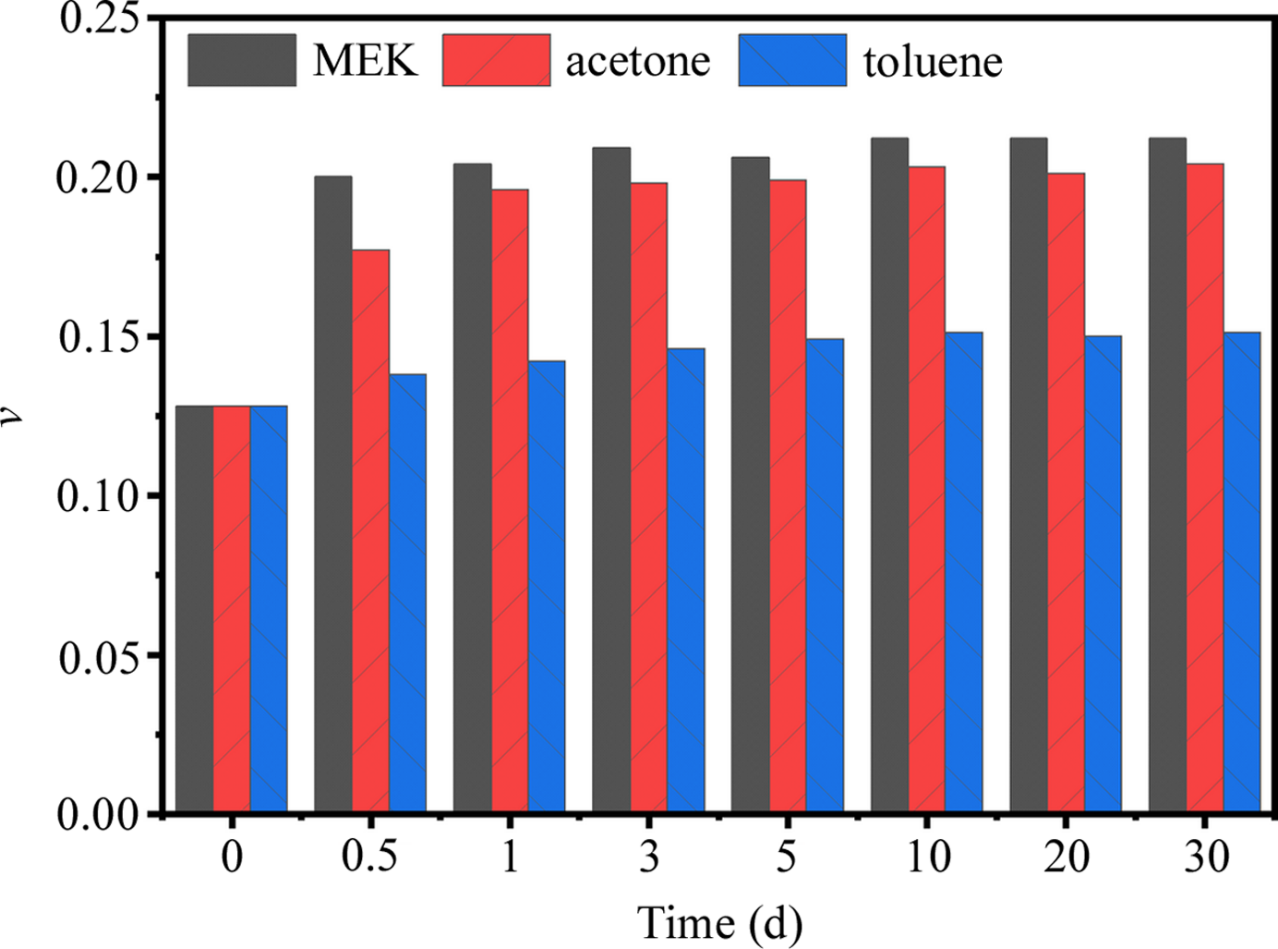
Hard-sphere volume fractions (*v*) as a function of solvent annealing time.

The fitted parameters are shown in Table 2 ▸. In line with our prior studies (Lin *et al.*, 2017 ▸; Tian *et al.*, 2018 ▸; Zhang *et al.*, 2019 ▸), the value of σ for PU samples generally falls within the range 0.2 to 0.4. In this study, we evaluated various initial values of σ and determined that 0.29 provides the optimal fitting performance. The median radius of HS domains (*R*_med_) moderately increased after exposure to the MEK vapor. In contrast, the annealing with acetone and toluene had a comparatively small effect on *R*_med_. Both MEK and acetone vapor annealing led to a minor increase in the hard-sphere interaction radius of the HS domains (*R*_HS_), whereas toluene annealing resulted in a slight decrease of *R*_HS_. The hard-sphere volume fractions (*v*) increased after solvent annealing with the MEK, acetone and toluene vapors. In other words, the interactions between the solvent molecules and PU macromolecular chains promoted a degree of microphase separation. The variation of *v* as a function of annealing time is displayed in Fig. 3 ▸. MEK vapor induced the most significant increase in microphase separation, with *v* rising from 0.126 (quenched state) to 0.218 (annealed for 10 d). According to the model fitting parameters, the order of influence exerted by the solvent vapors on the microphase structure of the PU was found to be MEK > acetone > toluene. This order is consistent with the adsorption capacities of the PU samples for the respective solvents (Fig. 1 ▸).

**Figure 4:**
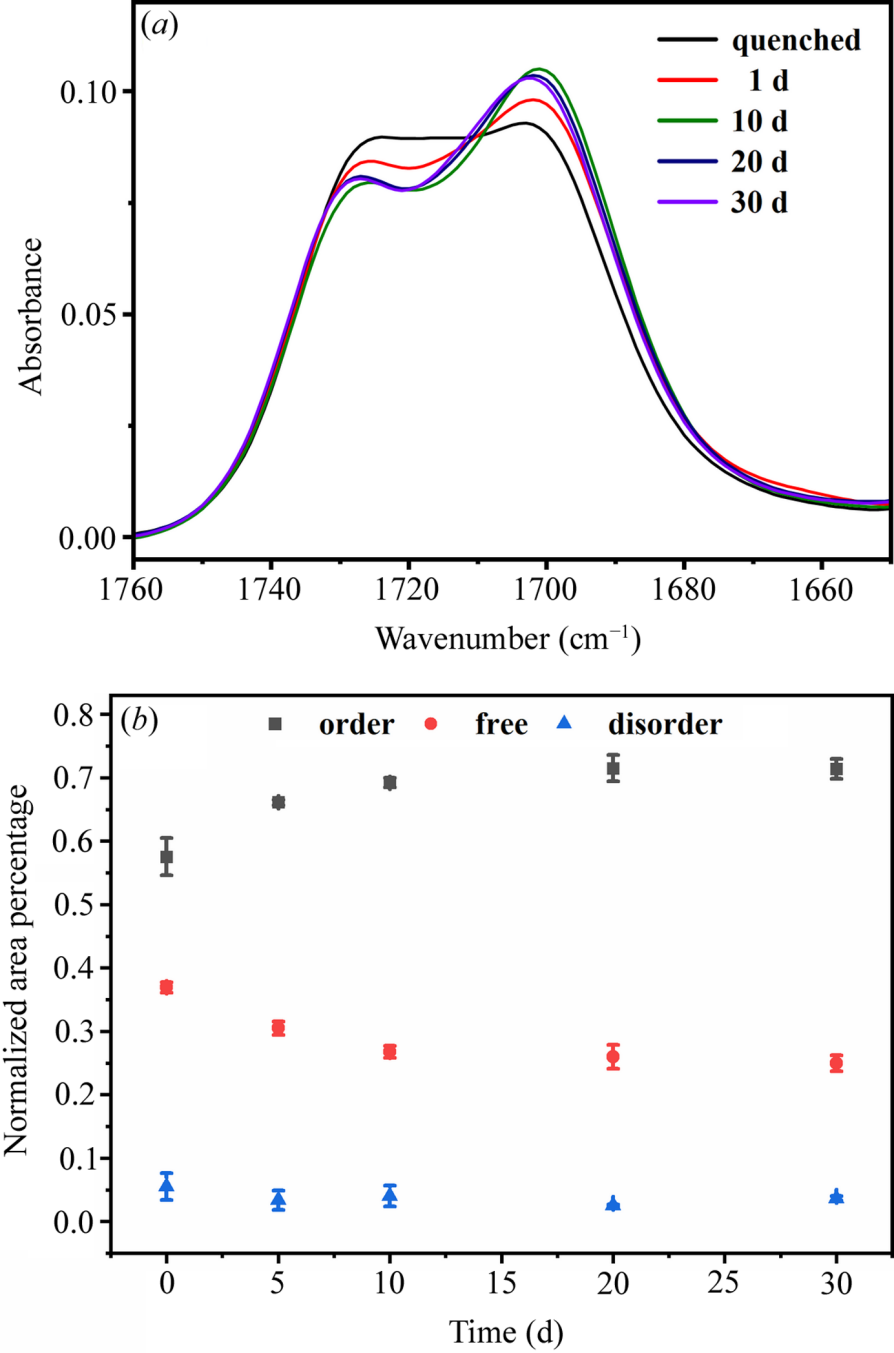
Variation of absorbance peaks in the C=O stretching vibrational region of FTIR spectra for MEK-vapor-annealed PU 1180A: (*a*) experimental data with aging times from 1 to 30 d; (*b*) fitted peak areas of the free, disordered and ordered C=O stretching vibration bands.

The full-range FTIR spectra obtained from the solvent-annealed PU samples are shown in Fig. S2. As a result of the solvent annealing, the intensities of the peaks attributed to the C=O vibrations changed: the peak at 1728 cm^−1^ decreased and the peak at 1701 cm^−1^ increased. The C=O stretching vibration region of the sample treated with MEK vapor was fitted to Gaussian–Lorentzian functions. As shown in Figs. 4 ▸(*a*) and S3, the C=O stretching vibration region is fitted by three peaks: free carbonyl (1728 cm^−1^), disordered hydrogen bonding C=O (1715 cm^−1^) and ordered hydrogen bonding C=O (1701 cm^−1^) (Coleman *et al.*, 1988 ▸; Ishihara *et al.*, 1974 ▸). The relative proportions of these three peaks were found to vary with the annealing times, as calculated using equations S1–S3. With the increase of annealing time using MEK vapor, the content of ordered hydrogen bonding C=O gradually increased, reaching a maximum (68%) at 10 d. Conversely, the amount of free C=O decreased [Fig. 4 ▸(*b*)], an indication that free C=O groups were transformed into ordered hydrogen-bonded C=O. This result implies that the dispersed MDI molecules in the PU matrix were aggregated to dense HS domains, in agreement with the increase of the hard-sphere volume fraction (Fig. 3 ▸).

### 3.3. Thermal stability

**Figure 5:**
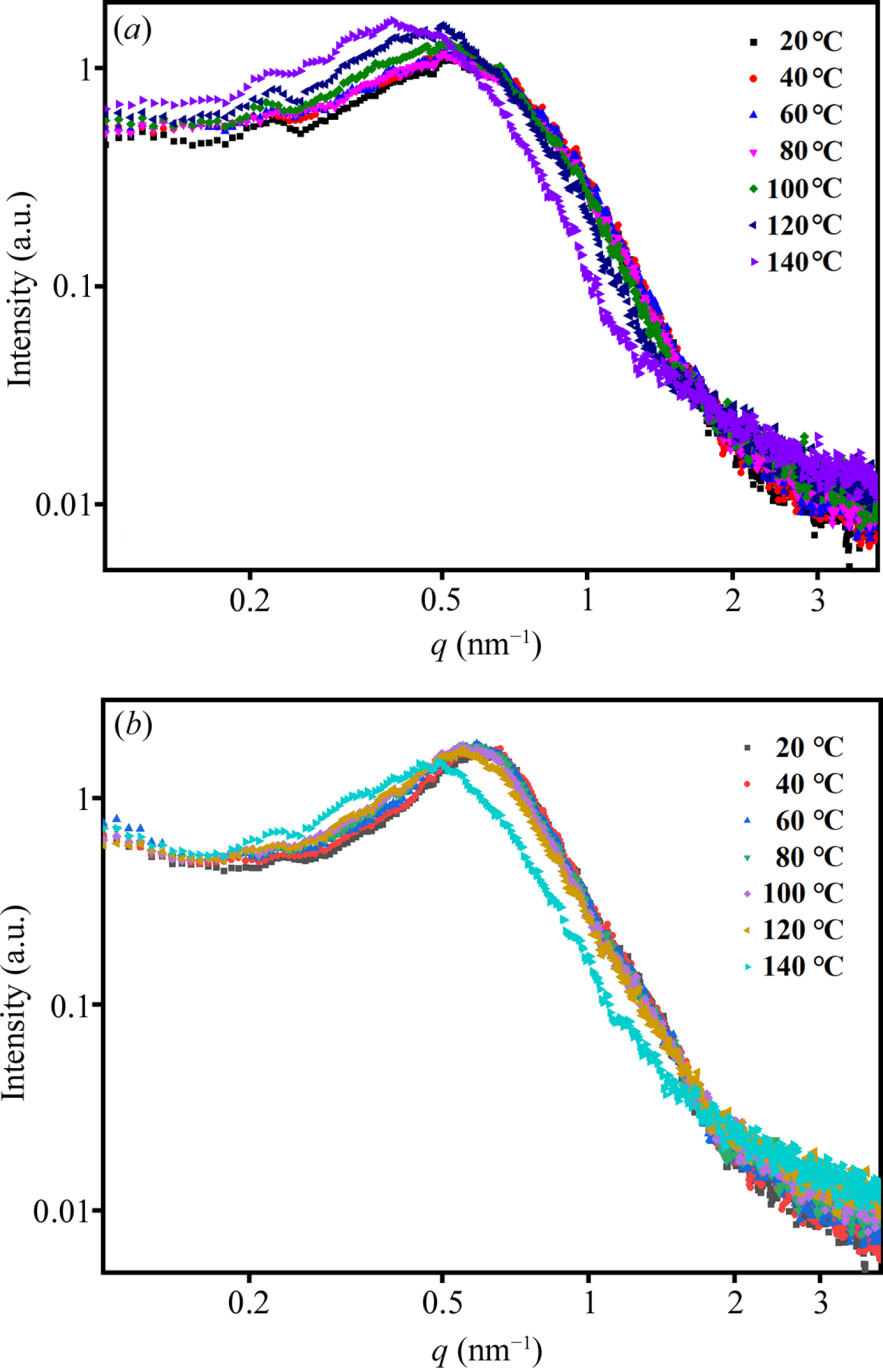
*In situ* variable-temperature SAXS data obtained from PU 1180A: (*a*) quenched, (*b*) MEK-vapor-annealed for 30 d.

**Figure 6:**
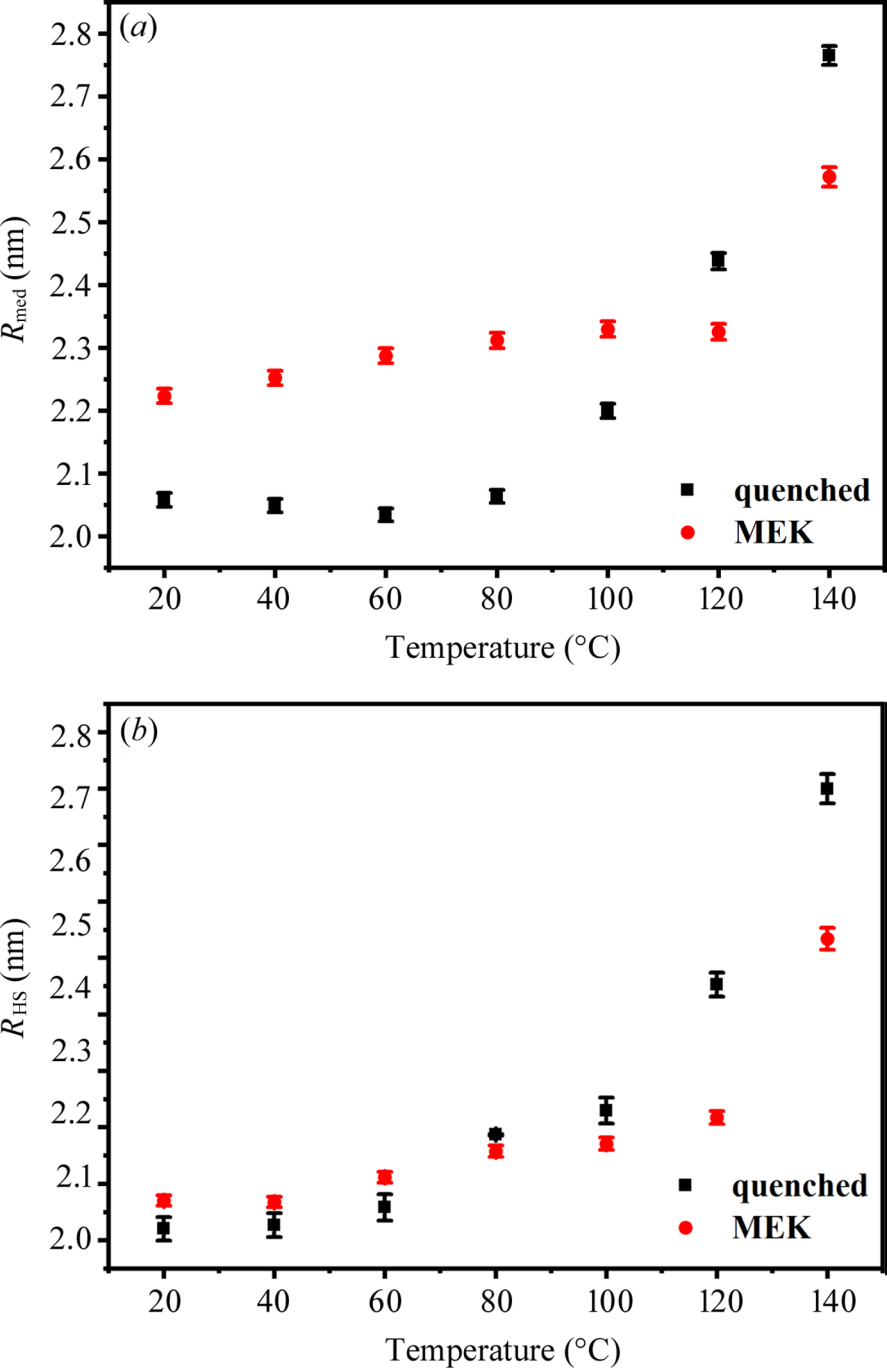
Parameters derived from the polydisperse hard-sphere model fitting for quenched and MEK-annealed PU 1180A: (*a*) median radius of HS domains (*R*_med_); (*b*) hard-sphere interaction radius (*R*_HS_).

The thermal stability of PU 1180A after MEK solvent annealing was investigated due to its significant impact on the microphase structure. The *in situ* variable-temperature SAXS data obtained from the quenched and MEK-vapor-annealed samples are shown in Fig. 5 ▸. The *in situ* variable-temperature SAXS data were fitted to the aforementioned model. The fitted parameters are shown in Fig. 6 ▸. The quenched PU samples exhibited a marked increase in *R*_med_ from 80°C, whereas the solvent-annealed PU showed significant changes in *R*_med_ at 120°C [Fig. 6 ▸(*a*)]. Correspondingly, the *R*_HS_ of the quenched PU increases rapidly at temperatures above 80°C, whereas the *R*_HS_ of the MEK-vapor-annealed PU demonstrates a gradual increase from 20 to 120°C, followed by a sharp increase beyond 120°C [Fig. 6 ▸(*b*)]. This observation suggests that the quenched sample contains a substantial number of unstable HS domains that readily dissociate and dissolve into the SS domains under thermal stimulation. In contrast, the MEK-vapor-annealed sample exhibits more stable HS domain structures with enhanced thermal resilience.

**Figure 7:**
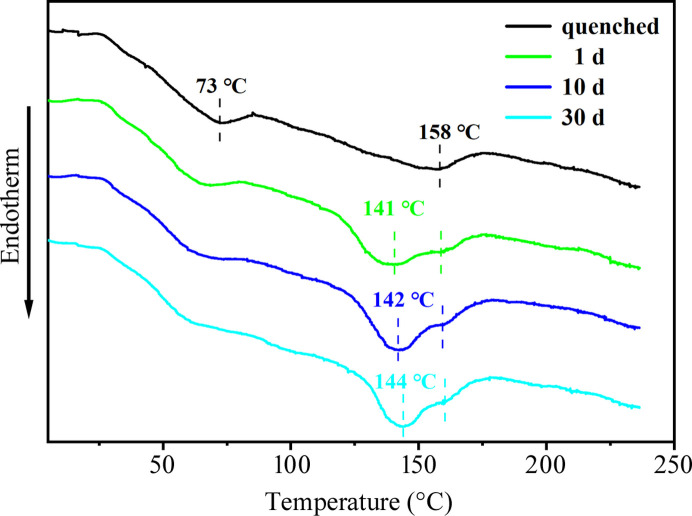
DSC data obtained from the quenched and MEK-vapor-annealed PU 1180A.

Fig. 7 ▸ shows that the quenched PU sample exhibits two typical heat absorption peaks at around 73 and 158°C, which are attributed to the destruction of loose and dense HS domains, respectively. After MEK solvent vapor annealing, a new endothermic peak appeared around 141°C, and this peak moved to 144°C with the extension of annealing time. These results provide support that solvent annealing induced the formation of ordered HS domains in the samples. This conclusion is consistent with the findings from the SAXS and FTIR analyses. In short, the MEK-vapor-annealed samples demonstrated superior thermal stability compared with the cold-water-quenched samples, attributed to the higher degree of microphase separation and a more stable HS domain structure in the solvent-annealed samples. The DSC data obtained from acetone- and toluene-vapor-annealed samples are shown in Fig. S4.

### 3.4. Discussion

**Figure 8:**
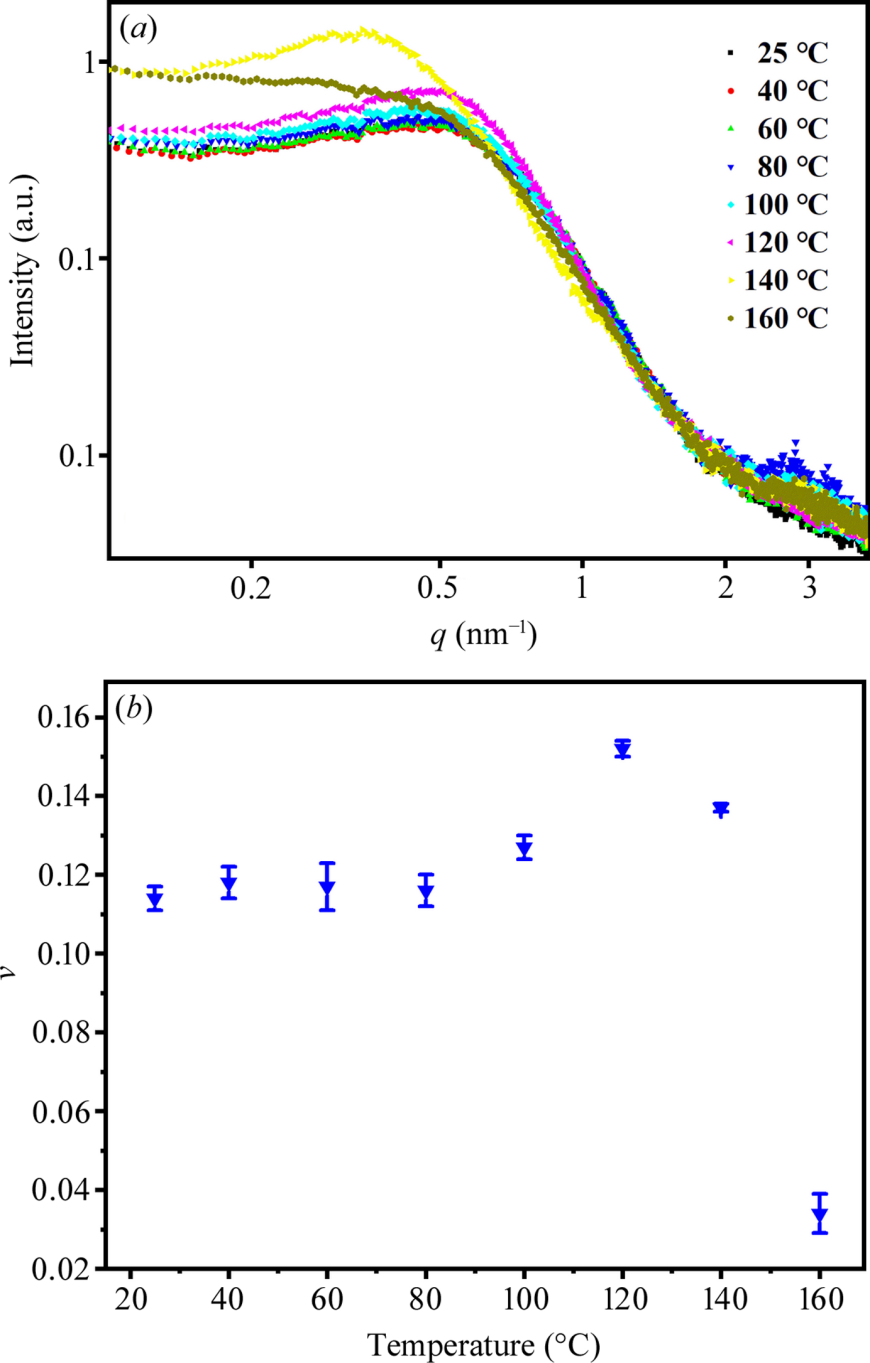
SAXS data obtained from the thermally annealed PU 1180A: (*a*) *I*–*q* curves; (*b*) hard-sphere volume fraction (*v*).

To compare the effects of solvent annealing and thermal annealing on the microstructure of PU 1180A, a set of samples with a 30 min annealing duration was prepared. The SAXS data of the thermally annealed samples are shown in Fig. 8 ▸. The *I*–*q* curves remained stable below 80°C. As the annealing temperature increased above 100°C, a significant increase in the scattering intensity was observed. The samples annealed at 120°C exhibited the strongest scattering intensity and the position of the interference scattering peak shifted towards small *q* values [Fig. 8 ▸(*a*)]. Following annealing at 160°C, the interference scattering peak vanished, indicating a substantial reduction in the density of the HS domains. Concurrently, the inflection of the scattering curve shifted toward a lower *q* range, suggesting a notable increase in the average size of the HS domains relative to samples annealed at temperatures below 120°C. The structural parameters fitted to the polydisperse hard-sphere model are presented in Table S1 of the supporting information. As shown in Fig. 8 ▸(*b*), the hard-sphere volume fraction (*v*) increases significantly above 80°C, reaching a maximum of 0.151 at 120°C, after which it decreases to 0.034 at 160 °C. According to the SAXS data and model fitting (Figs. 2 ▸ and 8 ▸; Tables 2 and S1), the differences between the thermal and solvent (MEK) annealing on quenched PU 1180A are as follows: *R*_med_ and *R*_HS_ increased moderately for solvent annealing, whereas *R*_med_ increased from 2.35 to 3.34 nm and *R*_HS_ increased from 4.81 to 7.16 nm on increasing the annealing temperature from 40 to 140°C. Notably, solvent annealing resulted in a higher *v* (0.22) than that (<0.16) obtained by thermal annealing.

**Figure 9:**
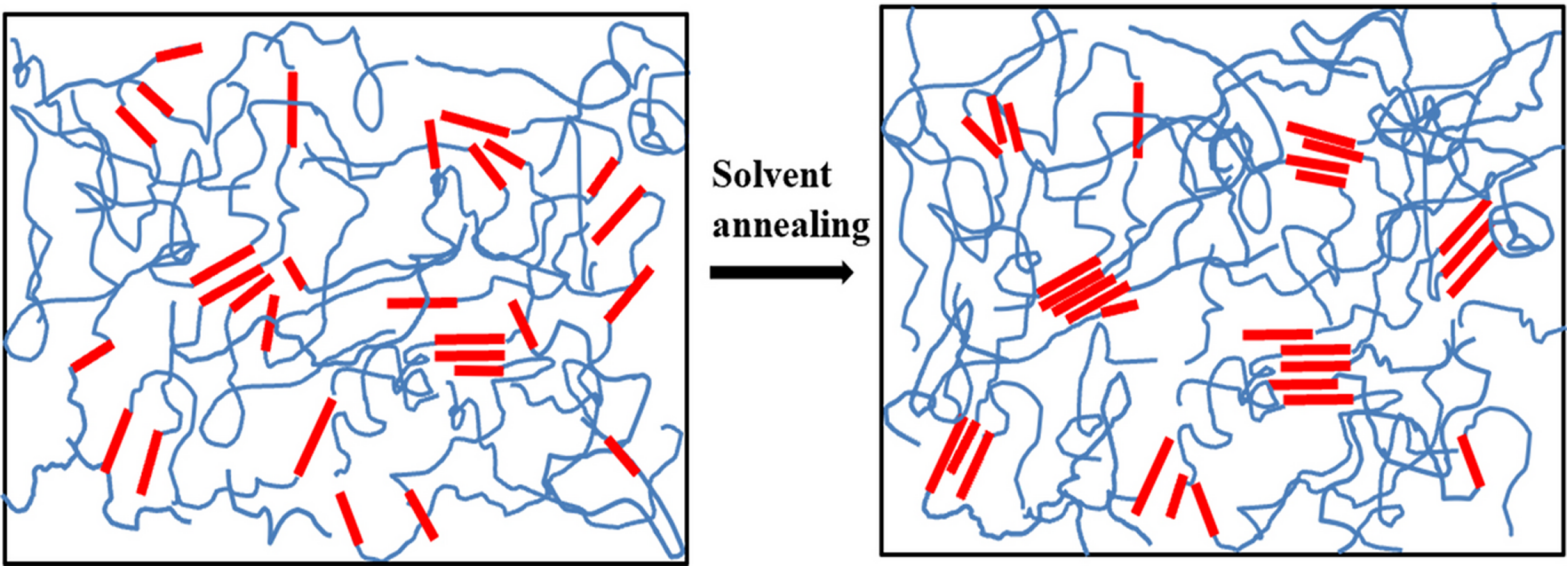
Schematic of microphase structural transformation in PU 1180A during solvent annealing.

The thermal annealing mechanism of PU 1180A aligns with established findings in the literature (Liu *et al.*, 2020 ▸, Orgilés-Calpena *et al.*, 2009 ▸; Saiani *et al.*, 2004 ▸; Kong *et al.*, 2019 ▸). The process is primarily driven by thermal energy, which induces the relaxation and subsequent rearrangement of polymer chains, thereby enhancing the microphase separation between HSs and SSs within the PU matrix. This thermally driven structural reorganization is significantly influenced by annealing temperature, duration and the intrinsic properties of the PU formulation. In contrast, solvent annealing operates through a distinct mechanism at ambient temperature, where the selective interaction of solvent molecules with both HSs and SSs governs the structural evolution of the polymer system. From the findings of this study, the insights into solvent annealing on PU 1180A are summarized as follows:

(1) The solvent molecules adsorbed to PU chains introduce a plasticization effect, which reduces the glass transition temperature of the polymer, increases the free volume of the polymer chains and lowers the energy barrier for segmental motion (*e.g.* rotation and vibration). Consequently, the HSs and SSs can rearrange more freely compared with the quenched state.

(2) From a thermodynamic perspective, the introduction of solvent molecules alters the free energy of the PU matrix. The adsorbed solvent molecules can disrupt the existing intermolecular interactions within the polymer, leading to a decrease in the enthalpic contributions associated with chain entanglements and hydrogen bonding. This disturbance allows for a transition to a more favorable thermodynamic state where the HSs and SSs can segregate more effectively.

(3) The choice of solvent plays a crucial role in this process. As shown in Fig. 1 ▸, the adsorption capacities of PU 1180A for MEK (450 mg g^−1^) and acetone (403 mg g^−1^) vapors are higher than that (331 mg g^−1^) for toluene, indicating that the organic solvent molecules with higher polarity (C=O) interact more effectively with the PU segments, promoting the phase separation. Compared with acetone, MEK has a larger molecular size and more complex molecular structure, which potentially enhances its interaction with PU chains, consequently leading to an increased degree of phase separation.

(4) Based on the SAXS results, a schematic of the microstructure evolution of the solvent-annealed PU is proposed, as shown in Fig. 9 ▸. The major effects of adsorbed solvent mol­ecules are to enhance the order of MDI packing in the existing HS domains and induce the aggregation of nearby dispersed HSs, leading to the formation of new HS domains.

(5) Solvent annealing can achieve a large degree of phase separation and enhanced thermal stability in the samples at room temperature compared with thermal annealing. In contrast, thermal annealing typically requires temperatures of 80°C or higher to achieve the desired microstructure (Saiani *et al.*, 2007 ▸; Pongkitwitoon *et al.*, 2009 ▸; Beniah *et al.*, 2016 ▸). Solvent annealing is particularly advantageous for sensitive materials and applications where excessive heat might cause degradation or undesirable changes.

## 4. Conclusions

The impact of solvent annealing on the microphase structure of PU 1180A was investigated as an alternative to high-temperature thermal annealing. The solvent molecules adsorbed to PU chains played a plasticization role, allowing the HSs and SSs to rearrange more freely compared with the quenched state. Among the solvents of MEK, acetone and toluene, MEK was outstanding for enhancing the degree of phase separation, as demonstrated by SAXS, FTIR and DSC results. *In situ* temperature-dependent SAXS analysis revealed that the solvent-annealed samples exhibited higher thermal stability compared with the as-quenched state. FTIR analysis confirmed that the free C=O groups were transformed into ordered hydrogen-bonded C=O, which is in agreement with the increase of hard-sphere volume fraction deduced by SAXS. Compared with thermal annealing, solvent annealing provides a more controllable and flexible approach to tailor the microphase structure of PU materials and to optimize their performance.

## Supplementary Material

Supporting figures and tables. DOI: 10.1107/S1600576725001633/ge5163sup1.pdf

## Figures and Tables

**Table 1:** Fitting parameters from the pseudo-second-order kinetics model for the adsorption kinetics curves of PU 1180A for different solvent vapors

Solvent vapors	*q*_e_ (mg g^−1^)	*k* (g mg^−1^ h^−1^)	*R* ^2^
MEK	450 (2)	0.0054 (3)	0.998
Acetone	403 (1)	0.0122 (5)	0.999
Toluene	331 (2)	0.0067 (5)	0.996
Water	19 (1)	0.12 (3)	0.937

**Table 2:** Structural parameters obtained from SAXS data of solvent-vapor-annealed PU samples by curve fitting using a polydisperse hard-sphere model The σ parameter was fixed at 0.29 throughout the fitting procedure.

Samples	*R*_med_ (nm)	*R*_HS_ (nm)	*v*
Quenched	2.05 (3)	4.64 (6)	0.126 (3)
MEK-0.5 d	2.50 (2)	4.78 (2)	0.190 (2)
MEK-1 d	2.33 (2)	4.67 (1)	0.201 (2)
MEK-5 d	2.46 (2)	4.83 (2)	0.206 (2)
MEK-10 d	2.38 (2)	4.87 (3)	0.218 (3)
MEK-20 d	2.46 (2)	5.02 (1)	0.218 (2)
MEK-30 d	2.48 (2)	5.07 (1)	0.218 (1)

Acetone-0.5 d	2.31 (3)	4.89 (3)	0.177 (1)
Acetone-1 d	2.32 (7)	4.70 (7)	0.196 (6)
Acetone-5 d	2.38 (4)	4.81 (1)	0.196 (1)
Acetone-10 d	2.41 (1)	4.81 (8)	0.203 (5)
Acetone-20 d	2.41 (2)	4.87 (2)	0.201 (1)
Acetone-30 d	2.34 (3)	4.77 (1)	0.204 (9)

Toluene-0.5 d	1.96 (1)	4.42 (2)	0.138 (1)
Toluene-1 d	2.05 (6)	4.57 (3)	0.142 (1)
Toluene-5 d	2.04 (2)	4.46 (3)	0.149 (3)
Toluene-10 d	2.05 (8)	4.41 (1)	0.154 (1)
Toluene-20 d	2.06 (7)	4.39 (7)	0.150 (3)
Toluene-30 d	2.07 (3)	4.40 (3)	0.152 (2)
