# Supplementary material for: Solvent-annealing-induced microphase separation in polyether polyurethane: a small-angle X-ray scattering study
Source: J Appl Crystallogr. 2025 Mar 19;58(Pt 2):564–72. doi: 10.1107/S1600576725001633 (PMC11957403; doi:10.1107/S1600576725001633)
Supplement: Supplementary file 1 [file j-58-00564-sup1.pdf]

JOURNAL OF APPLIED CRYSTALLOGRAPHY

Volume 58 (2025)

Supporting information for article:

Solvent annealing-induced microphase separation in polyether polyurethane: A small-angle X-ray scattering study

Shanshan Wang, Jiayao Song, Keping Chen, Mark Julian Henderson, Qiang Tian and Laszlo Almasy

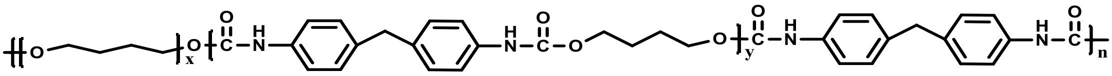

Figure S1 Chemical structure of the repeating units of PU 1180A.

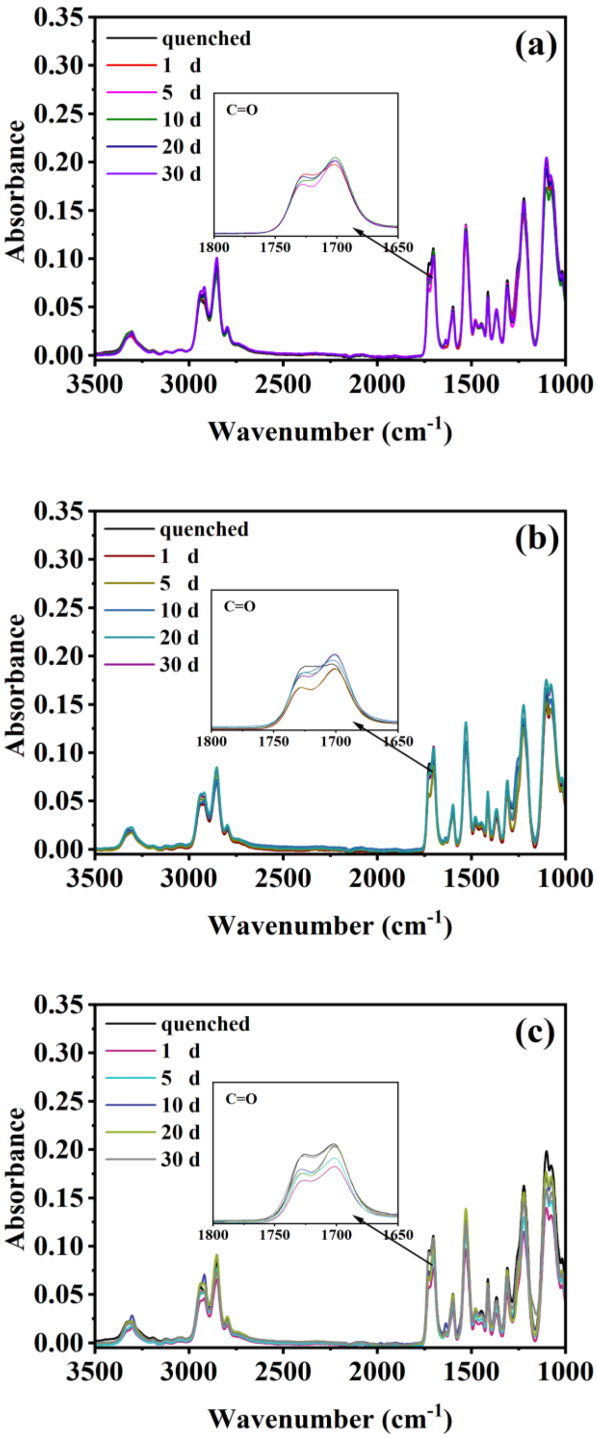

Figure S2 FTIR data obtained from solvents annealed PU 1180A using: (a) MEK; (b) acetone; (c) toluene.

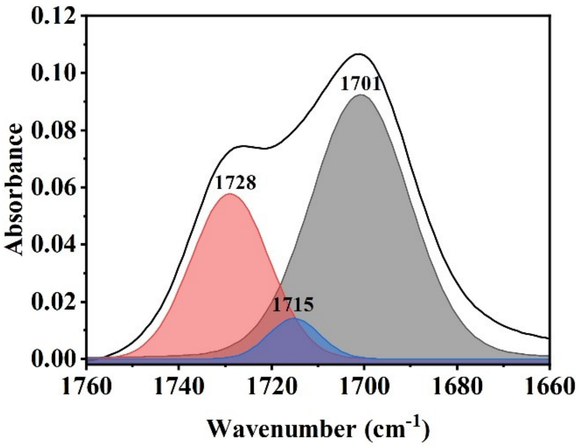

Figure S3 The fitting results for the C=O stretching vibration peaks of MEK vapor annealed PU 1180A.

The contents of free C=O (X_F), disordered hydrogen bonding C=O (X_D), and ordered hydrogen bonding C=O (X_O) were calculated from the ratios of the peak areas according to the following equations:

$$X_F = \frac{A_F}{A_O + A_D + A_F} \times 100\% \quad (S1)$$

$$X_D = \frac{A_D}{A_O + A_D + A_F} \times 100\% \quad (S2)$$

$$X_O = \frac{A_O}{A_O + A_D + A_F} \times 100\% \quad (S3)$$

where A_O, A_D, and A_F are the peak areas of ordered hydrogen-bonded C=O, disordered hydrogen-bonded C=O, and free C=O, respectively.

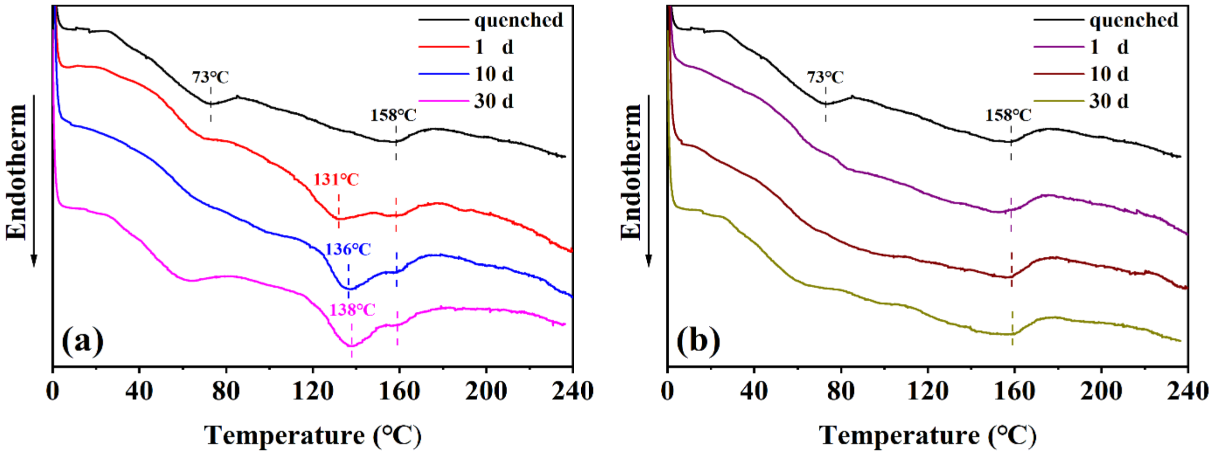

Figure S4 DSC data obtained from the acetone (a) and toluene (b) vapors annealed PU 1180A.

Table S1 Structural parameters of thermal annealed PU 1180A derived by SAXS data fitting to the polydisperse hard sphere model. The parameter σ was fixed at 0.29 throughout the fitting procedure.

Temperatures	R_{med} (nm)	R_{HS} (nm)	v
40 ℃	2.35(7)	4.81(1)	0.118(4)
60 ℃	2.36(1)	4.80(1)	0.117(6)
80 ℃	2.42 (1)	4.89(5)	0.116(4)
100 ℃	2.54(8)	4.96(8)	0.127(3)
120 ℃	2.66(6)	5.04(9)	0.151(2)
140 ℃	3.34(2)	7.16(7)	0.136(2)
160 ℃	2.9(1)	7.7(1)	0.034(5)
